# Neuroprotective Activity of Rasayana Formulation and Effect of Shigru Pallava Eye Drops on Intraocular Pressure as an Add-on Therapy Compared to the Standard of Care in Primary Open-Angle Glaucoma: Protocol for an Exploratory, Open-label, Two-arm Randomized Controlled Trial

**DOI:** 10.12688/f1000research.163722.1

**Published:** 2025-04-22

**Authors:** Deenadayal Devarajan, Manjusha Rajagopala

**Affiliations:** 1 Shalakya Tantra, All India Institute of Ayurveda, New Delhi, 110076, India

**Keywords:** Ayurveda, Glaucoma, Intraocular Pressure, Neuroprotection, Protocol, Rasayana, Shigru Pallava, Timira

## Abstract

**Background:**

Primary open-angle glaucoma (POAG) is a major cause of irreversible blindness worldwide, characterized by progressive optic neuropathy and loss of retinal ganglion cells (RGCs). Although lowering intraocular pressure (IOP) remains the mainstay of glaucoma management, many patients continue to experience vision loss, underscoring the need for adjunctive neuroprotective approaches. In Ayurveda, *Rasayana* therapies are believed to slow degenerative processes. One such intervention is a Rasayana Formulation (RF) comprising three oral preparations; Rasayana Churna (RC), Saptamrita Lauha (SL), and Yashada Bhasma (YB). Additionally, topical Arka (distilled extract) of Shigru Pallava (SP) has been used traditionally for glaucoma and may have an IOP-lowering effect.

**Methods:**

This is an open-label, two-arm, parallel-group randomized controlled trial with a 2:3 (control: intervention) allocation ratio. A total of 50 previously or newly diagnosed POAG patients (IOP < 30 mmHg) are planned for enrollment. The control group receives the conventional standard of care alone, whereas the intervention group receives standard care plus RF (2.5 g, taken orally twice daily) and SP eye drops (1 drop, four times daily), administered for 90 days with a further 90-day follow-up. Primary outcomes include changes in optic nerve function measured by visual field indices (mean deviation (MD), pattern standard deviation (PSD), visual field index (VFI)) and retinal nerve fiber layer (RNFL) thickness, while secondary outcomes include IOP changes.

**Results:**

Participant recruitment and data collection are ongoing. Final outcomes will be disseminated in peer-reviewed journals.

**Conclusions:**

If shown to be effective, the combined RF and SP eye drops could enhance neuroprotection and further control IOP in POAG, thereby addressing a significant need in current glaucoma therapy. This trial may provide a foundation for larger-scale investigations into integrative treatments for glaucoma management.

**Trial Registration:**

Clinical Trial Registry of India (CTRI) no. CTRI/2023/06/053681, dated 08.06.2023.

Available from: https://trialsearch.who.int/Trial2.aspx?TrialID=CTRI/2023/06/053681

**Protocol Version:** 3.0, dated 05.01.2023

## Introduction

Glaucoma is one of the leading causes of irreversible blindness worldwide, and its global prevalence is steadily increasing. ^1^ It is characterized by progressive degeneration of the optic nerve, resulting in corresponding visual field loss and eventual blindness if left untreated. Although the pathophysiology of glaucoma is multifactorial, open-angle glaucoma (OAG) is the most common clinical phenotype, defined by an open anterior chamber angle, characteristic optic nerve head changes, and progressive loss of peripheral vision followed by central visual field loss. ^2^ Elevated intraocular pressure (IOP) is a significant risk factor in OAG, though some patients may develop the disease without clinically elevated IOP. ^3^ Primary open-angle glaucoma (POAG), which lacks underlying secondary ocular disease, accounts for up to 75% of all glaucoma and remains one of the most prevalent and challenging forms to treat. ^4^ Current therapeutic approaches primarily aim to reduce IOP, as it is the only well-established method for slowing disease progression; however, multiple trials continue to evaluate novel diagnostic, management, and treatment strategies to address the multifactorial nature of POAG. ^5^

Despite the effectiveness of IOP-lowering therapies, many patients experience ongoing vision loss. ^6, 7^ Such observations highlight the need for neuroprotective strategies that either supplement or act independently of IOP reduction. Neuroprotection refers to interventions designed to prevent or delay glaucomatous neurodegeneration, chiefly by preserving retinal ganglion cells (RGCs). ^8^ In Ayurvedic medicine, diseases resembling glaucoma are correlated with “Adhimantha” and “Timira.” The Ayurveda concept of “Rasayana” signifies therapies that delay senile or degenerative changes, and “Chakshushya Rasayana” refers specifically to interventions intended to protect vision through supporting ocular tissues. By addressing both pathophysiological aspects and symptomatic presentations, these traditional approaches may offer additional benefits in glaucoma management.

POAG is now recognized primarily as an optic neuropathy, with specific pathophysiological features that differentiate it from other optic nerve diseases. ^9^ Although high-quality randomized controlled trials have demonstrated that lowering IOP can slow visual field deterioration, many patients still progress to blindness due to treatment limits or side effects. ^10, 11^ This has propelled further exploration of neuroprotective therapies independent of IOP lowering, focusing on maintaining RGC health. ^12– 14^ Preclinical studies have shown neuroprotective effects of various agents; yet, clinical investigations in humans remain inconclusive. ^15^ Memantine (an N-methyl-D-aspartate receptor antagonist) demonstrated benefit in other neurodegenerative diseases but showed no significant effect in glaucoma. ^16^ Brimonidine (an α2-adrenoreceptor agonist) exhibited potential in slowing visual field deterioration, though a controlled trial comparing 0.2% brimonidine with 0.5% timolol did not yield convincing evidence of neuroprotective efficacy. ^17^ Therefore, additional rigorous investigations into novel adjunctive therapies such as integrative Ayurveda-based interventions are warranted to address the remaining gaps in current glaucoma treatment.

Various Rasayana formulations are being used by Ayurveda practitioners in the management of ocular degenerative conditions such as glaucoma. The selected Rasayana Formulation (RF) in this study is a combination of three oral medicines: Rasayana Churna (RC), Saptamrita Lauha (SL), and Yashada Bhasma (YB). RC (an Ayurvedic herbal powder) has been traditionally used as a rejuvenating agent and is employed in a wide range of neurodegenerative conditions. ^18^ SL (an Ayurvedic herbal-mineral tablet) is a well-known classical formulation indicated in Timira (a term in Ayurveda used to describe certain stages of visual impairment). ^19^ YB (a purified, medicated nanoparticle preparation of zinc) is also indicated for eye diseases, as described in ancient texts of Rasa Shastra (Ayurvedic iatrochemistry), and is specifically referenced in the management of Timira. ^20^ Shigru Pallava (SP) Arka (distilled extract of *Moringa oleifera Lam.* leaves) is used as eye drops by Ayurvedic ophthalmic practitioners in patients of glaucoma. Some studies have already been conducted on the use of SP Arka in POAG. ^21, 22^ The juice of SP is mentioned by Acharya Vagbhata as a therapeutic option in conditions like Adhimantha. ^23^ The Arka (distilled) form of SP is also detailed in Arka Prakasha, a classical treatise on the therapeutic applications of distilled Ayurvedic preparations. ^24^

## Protocol

### Objectives

**Primary objective:** To compare the neuroprotective activity of a RF (2.5 g administered twice a day) as an add-on therapy to the standard of care at 30, 60, and 90 days in patients with POAG.

**Secondary objective:** To compare the effect of SP eye drops [1 drop (0.05 mL)/eye] four times a day on IOP when used as an add-on therapy to the standard of care at 30, 60, and 90 days in patients with POAG.

### Study design

This is an exploratory, open-label, two-arm, parallel-group randomized controlled trial with a 2:3 (control: intervention) allocation ratio. The total study duration for each participant is 180 days (90 days of intervention followed by 90 days of follow-up).

### Participants and eligibility criteria

All newly diagnosed and untreated patients of POAG will be considered for inclusion if they meet the following criteria: (1) POAG with IOP < 30 mmHg; (2) age between 30 and 60 years; (3) open irido-corneal drainage angle on gonioscopy; (4) documented glaucomatous visual field defects in at least one eye, accompanied by corresponding optic nerve head damage; and (5) at least two reliable visual field (VF) examinations in the year, defined by a false response rate below 15% and a detectable blind spot.

Patients were excluded if they had a visual acuity worse than 6/36 in the study eye, congenital/early childhood glaucoma, secondary glaucoma (exfoliative, pigmentary, or neovascular), anterior segment abnormalities affecting IOP measurement, or a visually significant cataract. Other exclusion criteria included ocular conditions that could cause VF abnormalities (e.g., degenerative myopia, diabetic retinopathy, maculopathy); best corrected visual acuity (BCVA) below 0.5 Snellen decimal fraction (> +0.3 LogMAR); severe VF loss (mean deviation (MD) worse than −12 dB in the better eye or −15 dB in the worse eye); inability to use topical medical therapy; concurrent treatment for another ophthalmic condition; previous ocular surgery other than uncomplicated cataract extraction, YAG laser capsulotomy, or laser glaucoma surgery (argon or selective laser trabeculoplasty); pregnancy or breastfeeding; medical unfitness for the trial; or participation in any other interventional research study.

### Diagnostic criteria

Patients were considered to have glaucoma if they presented with asymptomatic or nonspecific complaints such as headaches, frequent changes in presbyopic corrections, noticing blind spots or scotomas, or difficulty adapting to darkness and displayed at least two of the following three clinical indicators, with an open irido-corneal drainage angle confirmed on gonioscopy: (1) an IOP reading above 21 mmHg on more than one occasion, a diurnal IOP variation exceeding 8 mmHg, or an inter-eye IOP difference of more than 5 mmHg; (2) optic nerve head (ONH) changes suggestive of glaucomatous damage, including a vertical cup-to-disc ratio above 0.5, or a cup asymmetry greater than 0.2, neuroretinal rim thinning or notching, pallor, disc hemorrhages, or abnormal vessel patterns; and (3) visual field changes consistent with glaucomatous defects. In addition, a retinal nerve fiber layer (RNFL) defect extending from the ONH in a characteristic pattern, as viewed under red-free examination, supported the diagnosis.

### Settings and location

The study is conducted at the Eye Outpatient Department (OPD), First Floor, Hospital Block, All India Institute of Ayurveda (AIIA), New Delhi, India – 110076.

### Trial oversight committees

The trial is being coordinated at AIIA, New Delhi. The institute is the center for the overall coordination of the trial, including participant screening and enrollment logistics, oversight of data management processes, and communication with relevant regulatory authorities. The study protocol was first submitted for review to the Institutional Review Board (IRB), which assessed its ethical and scientific merit. Upon finding it suitable, the IRB granted provisional approval along with suggested revisions. After incorporating these recommendations, the revised protocol was forwarded to the Institutional Ethics Committee (IEC) for further evaluation. Following acceptance of the final modifications, the protocol received full approval for implementation. Additionally, the Department Research Committee (DRC) comprising the research guide, co-guides, advisors, and department heads monitor the trial’s conduct, oversees adherence to the protocol, addresses any protocol-related issues, and routinely assesses recruitment progress, data quality, and the need for any protocol amendments.

### Interventions

Participants are divided into two groups; details of the interventions are given in Table 1.

**Table 1.** Details of the interventions.

Group	Intervention	Route	Dose	Frequency	Duration
Group A	POAG Standard of Care (SOC) *	Topical/oral	As per AAO-PPP-G *	As required	90 days
Group B	POAG SOC	Topical/oral	As per AAO-PPP-G *	As required	90 days
	Rasayana Formulation	Oral	2.5 g	2×/day	90 days
	Shigru Pallava Eye Drops	Topical	1 drop (0.05 mL)	4×/day	90 days

* AAO-PPP-G refers to treatments recommended by the American Academy of Ophthalmology’s Preferred Practice Pattern Guidelines. g: gram, mL: milliliter, POAG: primary open angle glaucoma, SOC: standard of care.

**Shigru Pallava Eye Drops:** Each drop (0.05 mL) contains Arka (distilled aqueous extract) of Shigru ( *Moringa oleifera* Lam.). Participants instill one drop in each eye four times daily.

**Rasayana formulation:** This is a herbo-mineral combination provided as a 2.5 g dose consisting of powdered herbal ingredients and nanoparticles of minerals ( Table 2).

**Table 2.** Details and composition of Rasayana Formulation.

Sr. No.	Drug	Scientific/Botanical Name		Quantity
1	*Yashada Bhasma* (YB)	Zinc (Zn) nanoparticle		60 mg
2	*Saptamrita Lauha* (SL)	*Lauha Bhasma* (Iron (Fe) nanoparticle)		250 mg (comprising 50 mg of each constituent)
		*Yashtimadhu Churna*		
		*Triphala Churna*	*Haritaki Churna* ( *Terminalia chebula* Retz.)	
			*Vibhitaki Churna* ( *Terminali bellerica* Gaertn.)	
			*Amalaki Churna* * *(Emblica officinalis* Gaertn.)	
3	*Rasayana Churna* (RC)	*Guduchi* Churna ( *Tinospora cordifolia* Willd.)		2190 mg (comprising 730 mg of each constituent)
		*Gokshura Churna* ( *Tribulus terrestris* Linn.)		
		*Amalaki Churna* * *(Emblica officinalis* Gaertn.)		

* *Amalaki Churna* is a constituent of both SL and RC. Accordingly, a single 2.5 g dose of RF contains a total of 780 mg of fine *Amalaki* powder. mg: milligram, g: gram.

### Source of plant material and reagents

Shigru leaves were obtained from the in-house herbal garden of the AIIA. All raw herbal ingredients were sourced from reputable suppliers and authenticated by a botanist at the Regional Raw Drug Repository (RRDR), AIIA, under the Department of Dravyaguna Vigyana (Ayurvedic Materia Medica and Pharmacology). Yashada Bhasma and Lauha Bhasma were procured from a GMP-certified company (Manufacturing License No.: AYU-150). Both the SP drops and the RF were manufactured at the AIIA Pharmacy following in-house Standard Operating Procedures (SOPs) based on the Ayurvedic Formulary of India (AFI) and the Ayurvedic Pharmacopoeia of India (API), under the Department of Rasa Shastra and Bhaishajya Kalpana (Ayurvedic Iatro-chemistry). No chemical reagents (e.g., solvents or laboratory kits) were used in this study.

### Comparator

Conventional standard care of POAG, adhering to the American Academy of Ophthalmology - Preferred Practice Pattern Guidelines (AAO-PPP-G) recommendations, serves as the comparator. This may include topical eye drops (prostaglandin analogs, beta-blockers, etc.) or oral medications (e.g., carbonic anhydrase inhibitors) based on clinical judgment.

### Sample size

This is an exploratory study; no prior data exists for neuroprotection as the primary outcome in POAG with Ayurveda intervention. A minimum of 50 participants will be enrolled in total, with 30 participants assigned to the intervention group and 20 participants assigned to the control group.

### Randomization

A block randomization approach with variable block sizes is adopted to maintain balanced allocation across study arms at a 2:3 ratio. The random allocation sequence was generated by a research advisor using computer-based randomization software. Once a participant is confirmed eligible by the principal investigator (PI), enrollment will be documented in the study records, and the sealed, numbered, opaque, sequential envelopes (SNOSE) will be opened. The PI will then assign the participant to either the control or intervention group according to the concealed allocation contained within the envelope.

### Blinding

This is an open-label trial. Neither participants nor investigators are blinded due to the nature of the intervention.

### Criteria for discontinuing or modifying allocated interventions

If a participant experiences serious adverse events or shows clinically significant deterioration that appears linked to the RF or SP eye drops, the intervention may be paused or discontinued at the investigator’s discretion. Participants retain the right to withdraw voluntarily at any time. In cases where IOP or optic nerve parameters substantially worsen, or if an alternative therapy becomes necessary for emergent ophthalmic conditions, the intervention may also be modified or halted. If a participant becomes pregnant during the study, the intervention will be discontinued due to insufficient safety data in this population. All such modifications and discontinuations will be recorded in the case report forms (CRFs).

### Strategies to improve adherence to protocols

Participants will receive a compliance chart (see extended data) to track their daily intake of the RF and eye-drop usage. At each scheduled visit (30, 60, and 90 days), they will be required to return any empty or unused medication and eye-drop bottles, which will be counted to assess adherence. Regular check-ins via phone calls will be conducted to remind participants of follow-up appointments and dosing schedules (see extended data). During the baseline visit, each participant will be counseled on the importance of adherence, correct eye-drop instillation technique, and the potential benefits of completing the full intervention.

### Concomitant care and interventions

Participants will continue using standard anti-glaucoma medications, such as beta-blockers, prostaglandin analogs, or carbonic anhydrase inhibitors, as prescribed by treating ophthalmologists, unless contraindicated. Introduction of any new Ayurvedic formulation with overlapping ingredients or additional investigational drugs will be prohibited. Additionally, participants will be advised to maintain consistent dietary and lifestyle habits throughout the trial and to refrain from using any unauthorized herbal or nutraceutical supplements.

### Strategies for achieving adequate participant enrollment

A dedicated screening process for POAG is conducted at the Eye OPD, First Floor, Hospital Block, AIIA, focusing on patients over 30 years of age. Outreach efforts include distributing pamphlets on glaucoma and Ayurveda-based management to both visitors and patients at the institute. Additionally, flexible scheduling options, including six-day-a-week or after-hours appointments, are offered to accommodate working participants and increase enrollment.

### Plans to promote participant retention

Participants receive reminder phone calls and messages a week and a day before each scheduled follow-up visit. Those with scheduling conflicts are offered alternative appointment times to ensure flexibility. In cases where participants discontinue the intervention, the study team attempts to conduct outcome assessments such as measuring IOP, performing optical coherence tomography (OCT), and conducting VF tests to capture essential end-of-study data.

### Assessment and data collection

**Figure 1.**
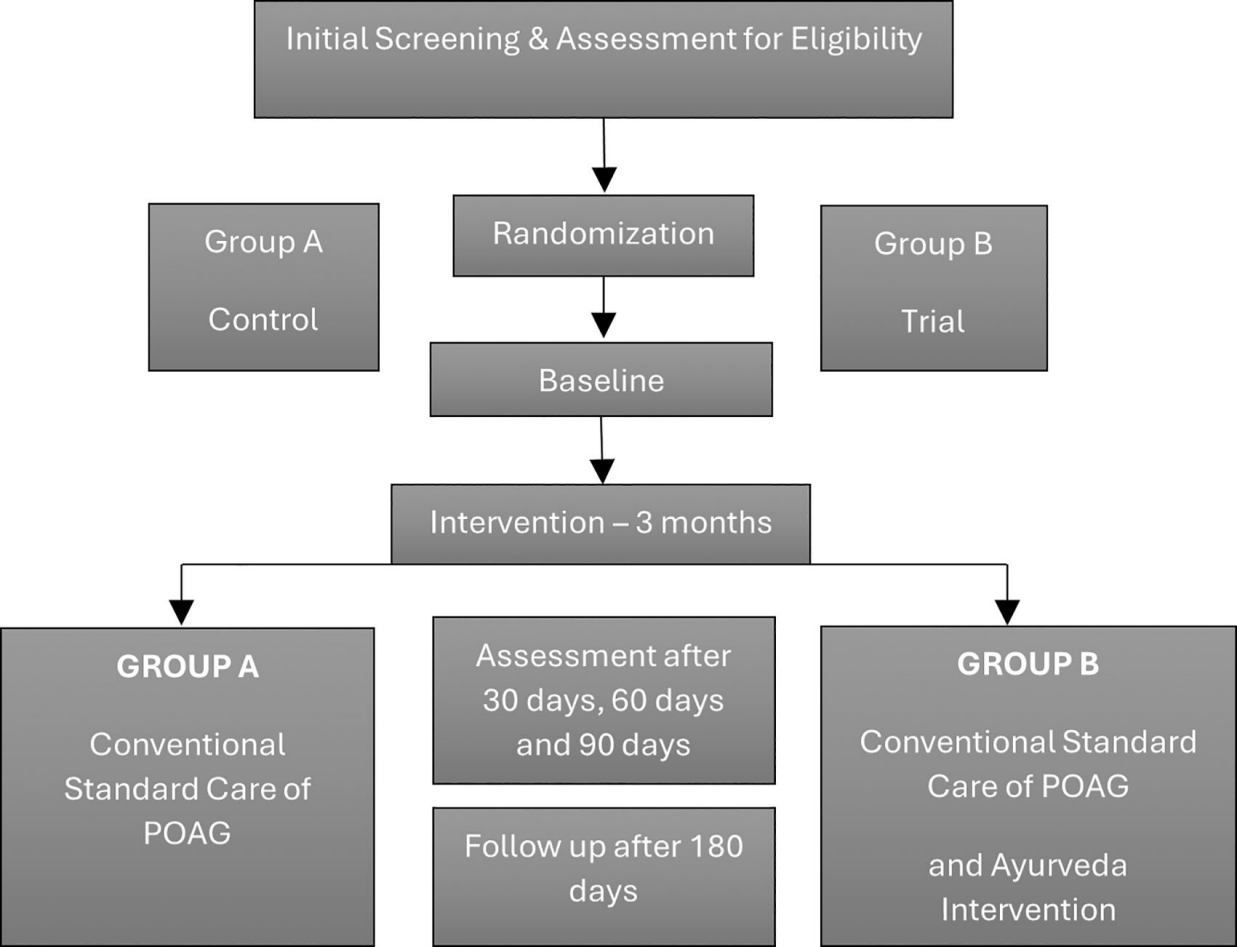
Activity chart. Study design flow chart illustrating the participant activity. Participants were screened and assessed for eligibility, then randomized into two groups: Group A (Control) and Group B (Trial). Assessments will be conducted in baseline, 30, 60, and 90 days during the 3-month intervention period, with a follow-up of 180 days.

The principal investigator (PI) oversees all data management activities, including data entry verification, cleaning, codebook maintenance, and data security. Participants will undergo baseline and follow-up evaluations at 30, 60, and 90 days (end of treatment), as well as at 180 days (90-day follow-up). Specific assessments include visual acuity (LogMAR), VF testing (perimetry), OCT RNFL thickness, IOP, CCT, and laboratory investigations (CBC, ESR, FBS, KFT, LFT), as outlined in Table 3. The overall schedule is detailed in Figure 1 and Table 4. Instruments such as the Humphrey Field Analyzer and OCT are recognized as valid and reliable for glaucoma diagnostics. All personnel responsible for VF tests, OCT scans, or tonometry received protocol-specific training. CRFs and compliance charts are securely stored at the coordinating center, and standardized operating procedures (SOPs) are employed throughout the data entry process to maintain consistency and accuracy.

**Table 3.** Details of parameter assessment.

S. No	Parameters		Unit	Instrument
1.	Visual acuity - Distant		Number in decimals	ETDRS LogMAR chart
				Landolt C LogMAR chart
				Tumbling E LogMAR chart
2.	Visual acuity - Near		Number in decimals	ETDRS LogMAR near vision chart
3.	Best corrected visual acuity		Number in decimals	ETDRS LogMAR chart
4.	Perimetry	GHT	Outside normal limits-1 Within normal limits-0	ZEISS Humphrey field analyser 3 (HFA), Mark III SITA standard 30–2
		VFI24-2	In %	
		MD30-2	dB	
		PSD30-2	dB	
5.	OCT- OPTIC DISC CUBE 200x200	Average RNFL Thickness	μm	ZEISS CIRRUS HD - Optical Coherence Tomography (OCT)
		RNFL Symmetry	%	
		Rim Area	mm ^2^	
		Disc Area	mm ^2^	
		Average C/D Ratio	Number in decimals	
		Vertical C/D Ratio	Number in decimals	
		Cup Volume	mm ^3^	
6.	IOP measurement		mm of Hg	Goldmann Applanation tonometer, TOPCON Non-Contact Tonometer (NCT), Adjusted value
7.	Central corneal thickness (CCT)		mm	TOPCON Pachymeter/ZEISS CIRRUS HD - OCT

CCT – central corneal thickness, C/D Ratio – cup-to-disc ratio, ETDRS – early treatment diabetic retinopathy study, GHT – glaucoma hemifield test, IOP – intraocular pressure, LogMAR – logarithm of the minimum angle of resolution, MD – mean deviation, NCT – non-contact tonometer, OCT – optical coherence tomography, PSD – pattern standard deviation, RNFL – retinal nerve fiber layer, VFI – visual field index.

**Table 4.** Participant timeline.

Parameter	Instrument	Screening	Baseline	30 days	60 days	90 days (End Tx)	180 days (Follow-up)
Informed consent	Consent form	+					
Demographic and medical history	Case report form	+					
Clinical examination	As per need	+	+				
Assessment of ADR	ADR reporting form			+	+	+	+
Assessment of drug compliance	Compliance chart			+	+	+	
Issue of trial drugs	Trial drug kit		+	+	+		
Visual acuity (distant & near)	ETDRS LogMAR Chart	+	+	+	+	+	+
Automated VF testing (30–2 SITA standard)	ZEISS Humphrey Field Analyzer 3 (HFA)	+	+	+	+	+	+
Retinal Nerve Fiber Layer (RNFL) Thickness	ZEISS CIRRUS HD-OCT	+	+	+	+	+	+
Intraocular Pressure (IOP)	Goldmann Applanation Tonometer/Non-Contact Tonometer (NCT)	+	+	+	+	+	+
Central Corneal Thickness (CCT)	TOPCON Pachymeter/ZEISS CIRRUS HD-OCT	+	+	+	+	+	+
CBC with ESR, FBS, KFT, LFT	Standard Clinical Laboratory Tests		+	–	–	+	–

ADR – adverse drug reaction, CBC – complete blood count, CCT – central corneal thickness, ETDRS – early treatment diabetic retinopathy study, ESR – erythrocyte sedimentation rate, FBS – fasting blood sugar, HFA – humphrey field analyzer, IOP – intraocular pressure, KFT – kidney function test, LFT – liver function test, LogMAR – logarithm of the minimum angle of resolution, NCT – non-contact tonometer, OCT – optical coherence tomography, RNFL – retinal nerve fiber layer, VF – visual field.

### Data management

Data entry is conducted by PI using verification procedures, with all information recorded in a password-protected electronic database. Each participant is assigned a unique study ID, and direct identifiers are stored separately from the clinical dataset. The electronic database is accessible only to authorized personnel, and all physical documents (CRFs) are kept in locked cabinets at the study site. To ensure data quality, built-in range checks and validation rules automatically flag any out-of-range or inconsistent entries (Microsoft® Excel® for Microsoft 365 MSO (Version 2503 Build 16.0.18623.20076) 64-bit).

### Statistical methods

Within each group, repeated-measures analysis of variance (RM-ANOVA) or a suitable post-hoc test will be employed to analyze continuous variables, including LogMAR acuity, visual field index (VFI), MD, pattern standard deviation (PSD), RNFL thickness, IOP, and central corneal thickness (CCT) depending on the normality of distribution. Between-group comparisons will be performed using the unpaired t-test or its nonparametric alternatives as needed. Laboratory parameters such as complete blood count (CBC), erythrocyte sedimentation rate (ESR), fasting blood sugar (FBS), kidney function test (KFT), and liver function test (LFT) will be assessed via paired t-tests for within-group changes and unpaired t-tests for between-group differences. All statistical analyses will be conducted using IBM SPSS Statistics (Version 29) or later.

The primary analysis follows the intention-to-treat (ITT) principle, incorporating all randomized participants who have received at least one dose of the allocated intervention. Missing data, when deemed missing at random, may be addressed using multiple imputation based on observed participant characteristics. A per-protocol (PP) analysis will also be performed among participants who strictly adhere to the intervention, serving as a sensitivity assessment to confirm the robustness of the results.

### Data monitoring

Given the exploratory nature of this trial and its relatively small sample size, a formal, independent data monitoring committee has not been established. Instead, an internal monitoring group (DRC) comprising guide, senior faculties from the AIIA periodically reviews data.

### Interim analyses

No interim analyses are planned due to the exploratory design and modest sample size. However, if substantial safety concerns emerge or a high incidence of serious adverse events appears to be associated with the study intervention, early termination may be considered.

### Adverse events reporting

Any adverse events arising during the clinical trial will be promptly reported to the National Pharmacovigilance Coordination Centre (NPvCC), AIIA, New Delhi, India ( https://www.ayushsuraksha.com). If any adverse event is determined to be related to the trial interventions, appropriate ancillary care and management will be provided to the affected participants as per protocol guidelines.

### Protocol amendments

All protocol amendments such as changes to the eligibility criteria or primary outcomes will be submitted to the IRB and IEC and updated accordingly in the CTRI. Formal notifications of these changes will be circulated among all investigators, trial registries, and relevant regulatory authorities. If any modification could affect participant safety or their willingness to remain in the study, participants will be promptly informed and given the opportunity to re-evaluate their continued participation.

### Confidentiality and data protection

Personal identifiers, such as names and addresses, are stored separately from the main clinical dataset to protect participant confidentiality. Any sharing of de-identified data with co-investigators or regulatory bodies will occur under secure data-transfer protocols. Physical copies of CRFs will be locked in secure cabinets, while digital records and databases will be password-protected. All study documents and electronic files will be retained for at least five years post-publication. Access to the de-identified final dataset will be restricted to the PI, co-PI, and the statistics team. No contractual agreement exists that would limit the research team’s right to publish or disseminate findings.

### Access to data

The complete study protocol will be made available upon reasonable request and may also be accessible through the CTRI registry. De-identified participant-level data may be shared with external researchers who submit a formal data-sharing proposal, subject to institutional approval. Syntax or scripts used for data analysis (e.g., SPSS code) may be provided to qualified investigators under a data-use agreement to safeguard participant privacy.

### Ancillary and post-trial care

Upon completing their participation, subjects will continue to receive standard ophthalmic care at the AIIA as appropriate. Any participant who suffers harm directly attributable to the trial interventions will be offered free medical treatment and compensation in accordance with institutional policies.

### Dissemination policy

The findings of this study will be disseminated through peer-reviewed journal publications and presentations at national and international conferences. The de-identified dataset supporting the results will be made available in an open-access repository.

### Authorship eligibility

All authorship decisions will adhere to the International Committee of Medical Journal Editors (ICMJE) guidelines, requiring substantial contribution to study design, data acquisition, data analysis or interpretation, manuscript drafting or revising, and final approval. No professional medical writers are employed; publications arising from the trial will be prepared by study investigators.

### Biological specimens

Since this trial does not require collecting biological specimens for genetic or molecular analyses, no samples will be stored for future investigations. Should any ancillary study requiring specimen collection be proposed, new ethical approval will be sought, and participants will be asked to give explicit, written consent for such purposes.

### Confounders

Potential confounding factors include the concurrent use of any Ayurvedic medications with overlapping active ingredients, dietary supplements containing zinc or other nerve tonics, and lifestyle choices such as smoking, alcohol consumption, or exposure to neurotoxic substances. These elements could influence outcome measures and must therefore be monitored and documented throughout the study.

### Limitations

The progression of neurodegeneration in POAG varies substantially among individuals, making small changes difficult to detect with precision. Moreover, because the RF and SP eye drops consist of multiple components, any observed benefits cannot be conclusively attributed to a single molecule or ingredient.

### Outcomes

The primary outcome is the neuroprotective activity of the study interventions, measured by changes in optic nerve function, including alterations in VF indices (MD, PSD, VFI) and RNFL thickness. The secondary outcome comprises IOP changes assessed via tonometry, evaluating the potential ocular hypotensive effect of SP eye drops.

### Study status

The trial is currently ongoing, with active participant recruitment and preliminary data collection in progress. Updated information about enrollment status and trial timelines will be reported in subsequent revisions or publications.

## Discussion

POAG is a chronic, progressive optic neuropathy marked by the degeneration of RGCs and their axons. This deterioration eventually leads to significant visual impairment if not treated adequately. Despite advancements that largely focus on lowering IOP, many patients continue to experience visual field progression, underscoring the need for additional therapeutic approaches, including neuroprotective strategies. Various risk factors, such as elevated IOP, older age, genetic factors, ethnicity, thin central cornea, diabetes mellitus, and vascular dysregulation, further complicate the multifaceted pathogenesis of POAG. ^25^ Although current standard-of-care practices successfully reduce IOP through pharmacological, laser, or surgical interventions, they can involve high costs, possible side effects, and a decline in quality of life due to ongoing VF loss. ^25^ This has fueled research into integrative therapeutic modalities that offer neuroprotection either independently or as an adjunct to IOP control.

From an Ayurvedic standpoint, POAG shares pathological and symptomatic parallels with the conditions known as Adhimantha and Timira. In Ayurveda, “Rasayana” therapies are known to delay or mitigate degenerative changes and fortify ocular function. Among such Rasayana preparations, the combination of RC *,* SL and YB has historically been utilized in the management of ocular degenerative diseases like cataracts and glaucoma. ^18– 20^ These herbal-mineral formulations are indicated for their potential neuroprotective effects and are deeply rooted in classical Ayurvedic texts. The Topical application of SP ( *Moringa oleifera* Lam.) eye drops is another Ayurvedic intervention documented by Acharya Vagbhata and has been supported by research exploring its efficacy in glaucoma. ^21– 23^ This approach possibly addressing the pathophysiological aspects Adhimantha, specifically targeting disrupted ocular circulation and tissue integrity. Therefore, combining these Ayurvedic modalities—oral RF and topical SP eye drops holds promise for neuroprotection and IOP management in POAG.

Preclinical research over the past two decades has investigated multiple neuroprotective agents, including brimonidine, memantine, and nicotinamide. Brimonidine has demonstrated potential in preserving RGCs, ^26, 27^ and memantine showed encouraging outcomes in animal models, ^28, 29^ though human trials have yet to replicate these benefits. ^30, 31^ Nicotinamide supplementation also appears promising, mitigating mitochondrial dysfunction and RGC death in mouse models susceptible to glaucoma. ^32^ However, clinical investigations focusing on neuroprotective strategies in modern medicine face challenges such as high dropout rates and limited efficacy, as evidenced by inconsistent results in brimonidine trials (e.g., LoGTS) and memantine’s failure to significantly slow glaucoma progression in phase III human studies. ^16, 30, 31^

Ayurvedic clinical trials have shown favorable outcomes when standard antiglaucoma regimens are supplemented with oral RF and SP eye drops. ^21, 22^ Improved visual acuity, better control of IOP, and enhanced VF have been observed. Given these encouraging findings, the present exploratory randomized controlled trial is designed to rigorously assess the neuroprotective capabilities of the specified RF, alongside the effect of SP eye drops on IOP, in patients with POAG who are already receiving conventional care. By systematically evaluating changes in IOP, RGC function, and structural markers such as RNFL thickness, this study aims to help bridge gaps in current knowledge and support the development of integrative, evidence-based treatments for glaucoma. If these methods demonstrate efficacy, future large-scale trials could further validate Ayurvedic interventions as a valuable adjunct to traditional glaucoma management.

### Research ethics approval

This study will be conducted in accordance with the principles expressed in the Declaration of Helsinki. The protocol received prospective approval from the IEC, AIIA, New Delhi, India (clearance no. IEC-308/19.12.2022/PhD-18/2022). Written informed consent will be obtained from each participant prior to enrollment, and all participant data will be maintained with strict confidentiality.

### Informed consent

Investigators will guide the informed consent process by providing both verbal and written explanations of the study in the local language. Participants will be encouraged to ask questions and will be given sufficient time to make an informed decision. Those who agree to participate will sign the consent form, in the presence of a witness (see extended data). No ancillary studies are planned in this protocol. If future studies necessitate collecting additional data or biological specimens, the research team will seek separate approval from the IRB or IEC, and participants will be asked to provide new, explicit consent for these ancillary components.

## Reporting guidelines

Zenodo: SPIRIT checklist - Neuroprotective Activity of Rasayana Formulation and Effect of Shigru Pallava Eye Drops on Intraocular Pressure as an Add-on Therapy Compared to the Standard of Care in Primary Open-Angle Glaucoma: Protocol for an Exploratory, Open-label, Two-arm Randomized Controlled Trial. https://doi.org/10.5281/zenodo.15188887. ^34^

Data are available under the terms of the Creative Commons Attribution 4.0 International license (CC-BY 4.0).

## Data Availability

No data is associated with this article. Zenodo: Dataset from an Exploratory Randomized Controlled Trial on the Add-on Effect of Neuroprotective Activity of Rasayana Formulation and Shigru Pallava Eye Drops on Intraocular Pressure in Primary Open-Angle Glaucoma. https://doi.org/10.5281/zenodo.15116362. ^33^
