# Supplementary material for: Neuroprotective Activity of Rasayana Formulation and Effect of Shigru Pallava Eye Drops on Intraocular Pressure as an Add-on Therapy Compared to the Standard of Care in Primary Open-Angle Glaucoma: Protocol for an Exploratory, Open-label, Two-arm Randomized Controlled Trial
Source: F1000Res. 2025 Apr 22;14:454. [Version 1] doi: 10.12688/f1000research.163722.1 (PMC12120413; doi:10.12688/f1000research.163722.1)
Supplement: Peer review history

# Reviewer report: Reviewer response for version 1

**DOI:** 10.5256/f1000research.180119.r380360

**Published:** 2025-05-29

**Authors:** Nasreen Hanifa

**Affiliations:** 1 Department of Shalya Tantra, Jaipur, India

This trial offers a promising, integrative approach to managing POAG by combining Ayurvedic and conventional therapies. While the concept is innovative and potentially impactful, the current limitations in study design and size must be addressed in future research to validate efficacy and scalability.

1. **Innovative Approach:** Incorporates traditional Ayurvedic medicine, potentially offering complementary benefits alongside standard care.
2. **Rigorous Design:** A randomized controlled trial design adds validity and structure, improving the reliability of results.
3. **Holistic Evaluation:** Looks at both structural (RNFL thickness) and functional (visual field indices) outcomes, giving a comprehensive view of disease progression.
4. **Potential Public Health Impact:** If effective, the approach could be a low-cost, accessible adjunct in glaucoma care, especially in regions where Ayurveda is widely practiced.

**Limitations:**

1. **Open-Label Design:** The lack of blinding may introduce bias in reporting and assessment of outcomes.
2. **Small Sample Size:** With only 50 participants, the trial may be underpowered to detect small but clinically significant effects.
3. **Short Duration:** A 90-day treatment and follow-up may be insufficient to capture long-term neuroprotective effects in a chronic disease like glaucoma.

Is the study design appropriate for the research question?

Yes

Is the rationale for, and objectives of, the study clearly described?

Yes

Are sufficient details of the methods provided to allow replication by others?

Yes

Are the datasets clearly presented in a useable and accessible format?

Yes

Reviewer Expertise:

Ayurveda Surgery, General surgery, Ano-rectal Surgeries, Gastro-intestinal disorders, lifestyle disorders

I confirm that I have read this submission and believe that I have an appropriate level of expertise to confirm that it is of an acceptable scientific standard.

# Reviewer report: Reviewer response for version 1

**DOI:** 10.5256/f1000research.180119.r380361

**Published:** 2025-05-28

**Authors:** Naveen B.S

**Affiliations:** 1 Sri Sri College of Ayurvedic Science and Research, Bangalore, India

- The rationale and objectives of the present study are well-defined and thoughtfully constructed. Primary open-angle glaucoma (POAG) remains a major global health concern due to its chronic progression and the irreversible nature of the vision loss it causes. Given its increasing prevalence and the limitations of current treatments, there is a pressing need to explore improved therapeutic strategies. This study seeks to investigate the potential benefits of introducing an adjunctive therapy alongside the standard treatment regimen. The objectives are clearly aligned with this rationale, aiming to assess whether the additional intervention can enhance clinical outcomes and better manage disease progression in POAG patients.

 

- The study design is well-suited to address the research question, which is clearly defined and appropriately constructed. The chosen design incorporates intervention assessments at 30, 60, and 90 days, providing a comprehensive evaluation over an extended period. This duration is sufficient to observe clinically meaningful outcomes, ensuring the robustness and reliability of the findings. The longitudinal nature of the study enhances its capacity to capture both short-term and sustained effects of the intervention, making it a strong and scientifically sound framework for a research protocol. Sufficient details of the methods provided to allow replication by others. 

 

- The methodology is described in sufficient detail to facilitate replication by other researchers, ensuring transparency and validation. Each step of the experimental and analytical processes is well-defined and scientifically structured, allowing for the study to be reproduced under similar conditions. The clarity and rigor of the methods enhance the credibility of the findings and lay a solid groundwork for future investigations. As such, this research may pave the way for further exploration and refinement of therapeutic strategies in the management of primary open-angle glaucoma (POAG).

 

- The datasets are presented in a clear, accessible format, allowing for straightforward interpretation and analysis. Their simplicity enhances usability, making them suitable not only for the current study but also as a valuable resource for future research on primary open-angle glaucoma (POAG). The structure and organization of the data lay a solid foundation for subsequent investigations aiming to build on these findings.

Is the study design appropriate for the research question?

Yes

Is the rationale for, and objectives of, the study clearly described?

Yes

Are sufficient details of the methods provided to allow replication by others?

Yes

Are the datasets clearly presented in a useable and accessible format?

Yes

Reviewer Expertise:

NA

I confirm that I have read this submission and believe that I have an appropriate level of expertise to confirm that it is of an acceptable scientific standard.
